# Efficacy of a Web-Based Safety Decision Aid for Women Experiencing Intimate Partner Violence: Randomized Controlled Trial

**DOI:** 10.2196/jmir.8617

**Published:** 2018-01-10

**Authors:** Jane Koziol-McLain, Alain C Vandal, Denise Wilson, Shyamala Nada-Raja, Terry Dobbs, Christine McLean, Rose Sisk, Karen B Eden, Nancy E Glass

**Affiliations:** ^1^ Centre for Interdisciplinary Trauma Research Faculty of Health and Environmental Sciences Auckland University of Technology Auckland New Zealand; ^2^ Department of Biostatistics and Epidemiology Faculty of Health and Environmental Sciences Auckland University of Technology Auckland New Zealand; ^3^ Ko Awatea Counties Manukau Health Auckland New Zealand; ^4^ Taupua Waiora Centre for Māori Health Research Faculty of Health and Environmental Sciences Auckland University of Technology Auckland New Zealand; ^5^ Department of Preventive and Social Medicine University of Otago Dunedin New Zealand; ^6^ Department of Medical Informatics and Clinical Epidemiology School of Medicine Oregon Health Sciences University Portland, OR United States; ^7^ Johns Hopkins Center for Global Health Johns Hopkins University Baltimore, MD United States

**Keywords:** eHealth, intimate partner violence, randomized controlled trial, New Zealand, depression, population groups

## Abstract

**Background:**

Intimate partner violence (IPV) is a human rights violation and leading health burden for women. Safety planning is a hallmark of specialist family violence intervention, yet only a small proportion of women access formal services. A Web-based safety decision aid may reach a wide audience of women experiencing IPV and offer the opportunity to prioritize and plan for safety for themselves and their families.

**Objective:**

The aim of this study was to test the efficacy of a Web-based safety decision aid (*isafe*) for women experiencing IPV.

**Methods:**

We conducted a fully automated Web-based two-arm parallel randomized controlled trial (RCT) in a general population of New Zealand women who had experienced IPV in the past 6 months. Computer-generated randomization was based on a minimization scheme with stratification by severity of violence and children. Women were randomly assigned to the password-protected intervention website (safety priority setting, danger assessment, and tailored action plan components) or control website (standard, nonindividualized information). Primary endpoints were self-reported mental health (Center for Epidemiologic Studies Depression Scale-Revised, CESD-R) and IPV exposure (Severity of Violence Against Women Scale, SVAWS) at 12-month follow-up. Analyses were by intention to treat.

**Results:**

Women were recruited from September 2012 to September 2014. Participants were aged between 16 and 60 years, 27% (111/412) self-identified as Māori (indigenous New Zealand), and 51% (210/412) reported at baseline that they were unsure of their future plans for their partner relationship. Among the 412 women recruited, retention at 12 months was 87%. The adjusted estimated intervention effect for SVAWS was −12.44 (95% CI −23.35 to −1.54) for Māori and 0.76 (95% CI −5.57 to 7.09) for non-Māori. The adjusted intervention effect for CESD-R was −7.75 (95% CI −15.57 to 0.07) for Māori and 1.36 (−3.16 to 5.88) for non-Māori. No study-related adverse events were reported.

**Conclusions:**

The interactive, individualized Web-based *isafe* decision aid was effective in reducing IPV exposure limited to indigenous Māori women. Discovery of a treatment effect in a population group that experiences significant health disparities is a welcome, important finding.

**Trial Registration:**

Australian New Zealand Clinical Trials Registry (ANZCTR): ACTRN12612000708853; https://www.anzctr.org.au/Trial/Registration/TrialReview.aspx?ACTRN=12612000708853 (Archived by Webcite at http://www.webcitation/61MGuVXdK)

## Introduction

Intimate partner violence (IPV) against women is a human rights violation with significant health consequences [1,2]. There is a substantial body of literature documenting the prevalence of violence against women; international evidence suggests that one in three women have experienced IPV or sexual violence [2]. The impacts of IPV on multiple aspects of health have been extensively documented—including mental health, sexual and reproductive health, and chronic conditions—and represent a significant health burden for women [3]. The World Health Organization Global Plan of Action calls for strengthening the role of the health system in addressing violence against women and girls [4,5], yet, there is a paucity of evidence testing theoretically informed interventions for women. In a Cochrane systematic review evaluating advocacy interventions providing safety planning or facilitating access to community IPV resources, 8 of the 13 studies recruited women from health care settings [6]. Although there were some benefits from brief advocacy interventions (ie, may provide short-term reduction in anxiety, distress, depression, and violence recurrence), there was significant heterogeneity among the studies leading to “uncertainty about the magnitude of beneﬁt and the impact of abuse severity and the setting.” In addition, a minority of women experiencing IPV access formal IPV services. For example, among women participants of the New Zealand Violence Against Women Study who had ever been physically or sexually abused by a partner, 69% (658/956) had never told a formal resource about their partner’s behavior [7].

Electronic health (eHealth) provides an opportunity to reach a broad population of women and deliver an interactive, tailored intervention at no cost, at any time of day, and free from the stigma that may be associated with face-to-face interventions. A novel Web-based safety decision aid for women experiencing IPV was recently developed in the United States [8]. The decision aid, informed by an empowerment model [9] and decision-aid science [10], includes priority-setting activities, risk assessment with feedback, and tailored safety planning. In a randomized controlled trial (RCT), women’s decisional conflict reduced after a single use of the eHealth intervention [11]. To build further evidence, we conducted a concurrent replication of the US trial regionalized for Aotearoa New Zealand culture [12]. The New Zealand (*isafe*) trial advances the US trial in providing fully automated Web-based trial recruitment, eligibility screening, and consent. In this RCT, we tested the efficacy of an interactive Web-based safety decision aid. We hypothesized that the decision aid would improve mental health and reduce IPV exposure.

## Methods

### Trial Design and Participants

Our study protocol is described elsewhere [13]. Briefly, the *isafe* study consisted of a two-arm parallel RCT of a Web-based safety decision aid for women experiencing IPV. Efficacy of the decision aid was assessed primarily using participants’ exposure to IPV and mental health after 12 months of access to the decision aid. Participation was open to English-speaking women aged 16 years or older in New Zealand who reported exposure to current IPV. They also needed access to a safe email address (to which only they had password-protected access) to send and receive study-related information. The trial was approved by the Auckland University of Technology Ethics Committee (reference number 12/51).

Complete details regarding participant recruitment and engagement can be found elsewhere [14]. Participants enrolled in the study by accessing a secure New Zealand registration website; the most common referral source was a Web classified advertisement. A participant was considered enrolled once they had visited the registration website and (1) met eligibility criteria, (2) consented to participate, (3) provided contact information, and (4) passed a validation check. The validation check to reduce the risk of fraudulent enrollment involved automated matching against the New Zealand electoral roll (based on name and address) or a manual process of logic checking against information available from Web searching.

### Randomization and Masking

Once enrolled, participants were randomized by software to the control or intervention arm. Randomization was based on a minimization scheme. Two stratification factors (severity of violence and children) and two random factors each with two equiprobable levels were used to achieve the minimization. Severity of violence factor was dichotomous, based on one positive response to the IPV eligibility items versus two or more. The children factor was also dichotomous, identifying whether the participant had one or more children versus none.

**Figure 1:**
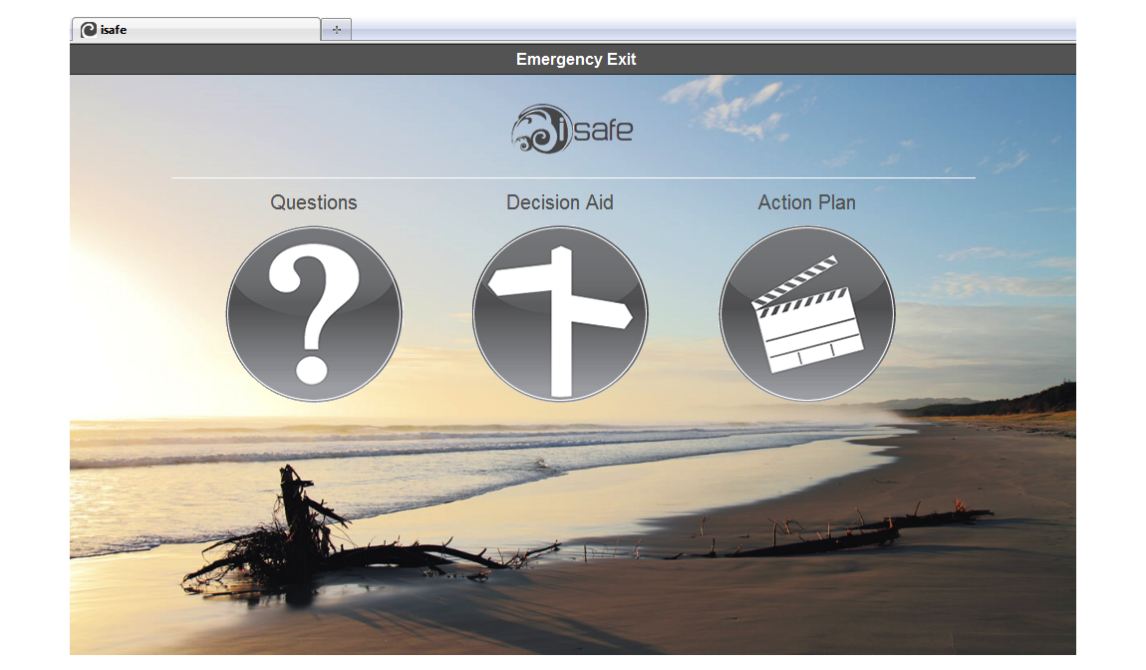
Screenshot of isafe navigation.

After randomization, participants were sent an email with a username, password, and URL access for the website. The participant’s allocation was kept secret from herself and the study team in New Zealand. There was no procedural difference between arms until after baseline measures were obtained. After baseline measures collection, participants in different arms were exposed to different screen contents, corresponding to the control and active interventions described below (see eg, Figure 1).

### Procedures

Women randomly assigned to the intervention group were able to access a Web-based safety decision aid throughout the 1-year postbaseline follow-up period via the secure password-protected trial website. The decision aid intervention included three components. The first component was a safety priority setting activity based on a multicriteria decision model developed in the United States [8]. The five criteria (priorities) were evaluated for the New Zealand context [12] and minor wording changes made to the descriptions [13]. Women moved a sliding bar toward the priority that was most important to them for all pairwise combinations. Through a series of matrix computations using the analytic hierarchy process [15], the program provided feedback to the participant summarizing her priorities. The second component was the Danger Assessment (DA) [16] or Danger Assessment-Revised (DA-R; for female same-sex relationships) [17]. Women completed the DA or DA-R and received immediate scored feedback on their level of danger for severe or lethal violence in the intimate relationship, ranging from variable danger to extreme danger. The third and final component was an interactive process using an underlying matrix of resources to help women develop an individually tailored action plan. The matrix included local, regional, and national resources; and tips about safety for the participant and her children based on her safety priorities and DA or DA-R scores.

Women randomly assigned to the control group were able to access a standardized list of resources and a standardized emergency safety plan throughout the 1-year postbaseline follow-up period via the secure password-protected trial website. They were not provided with individualized feedback or tailored action plan.

Participants were followed up for 1 year following completion of their baseline measures. Assessments were scheduled to take place at baseline, then 3, 6, and 12 months after completion of baseline. Women were invited to complete assessments at each time period regardless of prior assessment completions. Retention mechanisms included automated emails sent to participants at 1, 2, 4, 8, and 10 months. If a participant was 6 weeks late for a scheduled follow-up visit, the research team sent email or phone reminders (following the woman’s safety instructions).

Logs were maintained by study personnel documenting phone and email contacts with participants, potential unanticipated events, and other issues affecting the trial (such as server interruptions). Study safety protocols addressed computer safety procedures; confidentiality measures; support and referral procedures in case of violence or distress, including suicidality; responding to calls to the study site by partners or children; and investigation and reporting of unanticipated events. In addition to safety review during data monitoring committee (DMC) meetings, study logs and media reports of serious IPV events were monitored during the trial. The study protocol outlined the safety review process, which for study-related serious adverse events would involve escalation to the principal investigator and chairs of the ethics and data monitoring committees and reporting to the funder and university legal counsel.

### Outcomes

The mental health primary outcome consisted of depression measured by the Center for Epidemiologic Studies Depression Scale-Revised (CESD-R) [18-20] at 12 months. The CESD-R score, ranging from 0 to 80, was used. Higher scores are associated with more depression symptomatology, with scores of at least 16 indicative of depression. The IPV exposure primary outcome consisted of the Severity of Violence Against Women Scale (SVAWS) [21] at 12 months. For the SVAWS we used a 1 (never) to 4 (many times) subjective frequency scale for all items; total SVAWS score ranges from 46 to 196, with higher scores associated with greater exposure to IPV. Secondary violence domain outcomes included SVAWS total score at 3 and 6 months, SVAWS subscales (threats of violence, acts of violence, and sexual violence), and Women’s Experience with Battering (WEB) [22]. Secondary mental health domain outcomes included CESD-R score at 3 and 6 months, Post-Traumatic Stress Disorder Checklist-Civilian Version [23,24], Alcohol Use Disorder Identification Test dichotomized [25,26], and Drug Abuse Screening Tool [27,28]. Decisional process secondary outcome was measured using the Decisional Conflict Scale [29], safety-seeking behavior using the Safety Checklist [30,31], and Safety Checklist Helpfulness. Assessment schedules and time points are included in Multimedia Appendix 1.

### Statistical Analysis

As described in the published protocol [13], the figure of 340 women accounts for an upper limit of 35% dropout by 12 months based on attrition rates for previous Web-based studies (eg, the Recovery via Internet from Depression trial). Results from a New Zealand trial support the planned study to have 80% power to detect a 37% reduction in depression (CESD-R). Data from an international IPV study indicated that the proposed study would have power superior to 80% to detect a difference of −11.2 in IPV exposure (corresponding to ≈ ½ standard deviation baseline Severity Violence Against Women Scale).

Plans for all inferential analyses were finalized before allocation unblinding in a full statistical analysis plan. Although randomization was technically carried out before baseline assessment, the allocation remained entirely concealed until all baseline information was obtained. Accordingly, the intention-to-treat (ITT) analysis set consisted of all participants who provided data at baseline on a primary outcome. The main analyses were carried out in a modified ITT analysis set, consisting of the ITT analysis set from which participants with no postbaseline assessments were excluded. Sensitivity analyses were carried out in the ITT set to assess the effect of this exclusion on treatment effect estimates [32]. A per-protocol analysis set was also defined, excluding from the modified ITT set any participant identified with an eligibility violation (such as from repeat enrollment) or a major protocol violation (not receiving the intervention) and assigning the participant to the allocation arm corresponding to her actual uptake of the intervention.

All postbaseline assessments for a given outcome were entered in the regression models, initially assumed to be normal with a participant-specific random effect, adjusted for the baseline value, other covariates as described below, and placing the intervention in interaction with the assessment time, taken as a factor. A blind review, absent any information regarding allocation, was undertaken for each outcome for an assessment of missingness, a visual assessment of residual normality, an assessment of the covariance structure of the repeated measures, and an assessment of candidate covariates (ethnicity, children, paid employment, and age group) to include in the final analysis. In a model including all covariates, assessment time but no information regarding allocation, only covariates achieving a partial coefficient of determination larger than 1% were retained to adjust the final model. Subgroup analyses regarding children in their care versus not and Māori ethnicity versus not were planned for the primary outcomes. These proceeded along the same model as above, including a three-way interaction between treatment, assessment time, and subgroup indicator. Although women could select multiple ethnicities, a priori hierarchy prioritized Māori ethnicity.

Missing data at baseline was imputed using the mode of the variable in the observed ITT values [33]. Missingness in both primary outcomes was found to be significantly related to the last observed value of the outcome in question, to the assessment time being at 12 months in the case of CESD-R but to no other baseline variable, indicating that adjusting for baseline and fitting the available repeated measures with a suitable (nondiagonal) covariance structure appropriately removed the risk of bias from missingness under a missing at random assumption [34].

The sensitivity analyses to be carried out in the full ITT set consisted in the production of point estimates only with missing values singly imputed at each assessment time according to four different schemes: (1) ITT extension: missing values because of attrition imputed as the mean of the observed values in the control arm; missing values from nondropouts imputed as the mean of the arm to which the participant was allocated; (2) Return to baseline: all missing values imputed as the baseline value; (3) Worst case for intervention: missing values imputed as the worst intervention arm outcome in the intervention arm, and the best control arm outcome in the control arm; and (4) Best case for intervention: missing values imputed as the best intervention arm outcome in the intervention arm, and the worst control arm outcome in the control arm.

Mediation analyses based on multivariate regression were to be completed only if the intervention proved to be significantly related to the hypothesized mediators at a 0.15 level. All other tests of hypotheses were carried out using a significance level of 0.05 and two-sided alternatives. There was no adjustment for multiple testing. All analyses were carried out in the R software environment, version 3.1 (R Foundation for Statistical Computing, Vienna), using packages *lme4* [35] and *nlme.* An independent DMC was formed and met for the first time in September 2013, thereafter meeting four more times at approximately 6-month intervals. The role of the DMC was detailed in a charter.

## Results

### Participants

**Figure 2:**
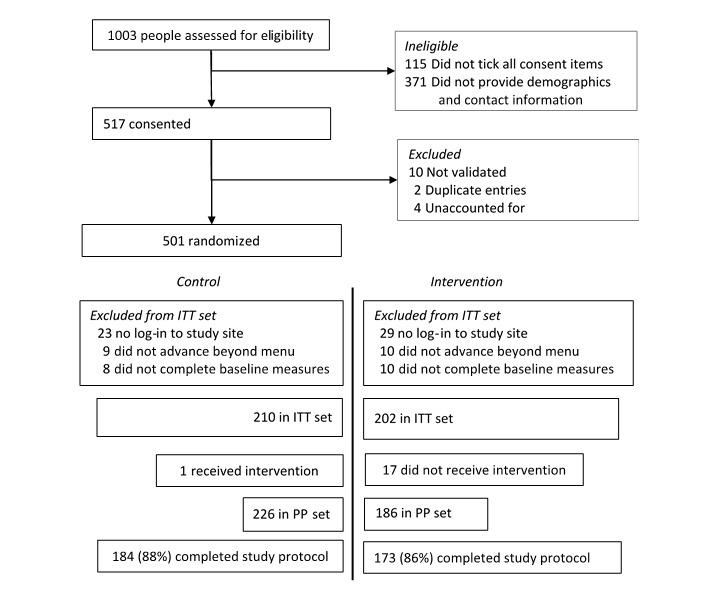
Trial profile (ITT=Intention to treat; PP=Per-protocol).

The 412 participating women (Figure 2) were typically young (interquartile range [IQR]=23, 36 years) and lived in a main urban area (83%); 45% (185/412) were responsible for the care of one or more children living in the household, 40% (165/412) were in paid employment, 27% of participants self-identified as Māori compared with 12% among women 15 years of age or older living in New Zealanders (2013 census, NZ Stats table), and 30% (124/412) of participants lived in neighborhoods in the highest deprivation quintile compared with the expected 20% (82/412).

### Baseline Characteristics

At baseline, partners were often living with the participant (227/412, 55%). Women’s decisional conflict about their partner relationship was evident: half of the participants were “unsure” of the future of their relationships, with one-quarter planning to end the relationship and one-quarter planning to remain in the relationship. Most women accessed the *isafe* study on a computer in their home (259/412, 63%) or the home of a friend or family member (37/412, 9%). The median SVAWS score was 84 (IQR 67, 103). The median CESD-R score was 32 (IQR 18, 50). A large proportion of *isafe* participants (309/412, 75%) evidenced depressive symptoms (score greater than 16); one in 12 reported suicidal thoughts (“I wished I was dead”) “nearly every day” in the prior 2 weeks.

Baseline characteristics, including violence severity and depression, were similar for women allocated to control or intervention (Table 1). The known inequities of race and socioeconomic deprivation were evident among our study participants: among the 113 Māori participants, 42.5% lived in the highest deprivation quintile; this compared with 25.1% among the 299 non-Maori participants. Although Māori were overrepresented in high deprivation neighborhoods, deprivation was not associated with group or outcome (and thus, not a confounder). Seventy-five percent of participating women completed all three follow-up assessments (73% of women assigned to control group and 76% of women assigned to intervention group). Retention rates at 3, 6, and 12 months were 81%, 83%, and 87%, respectively.

**Table 1:** Baseline characteristics of the intention-to-treat analysis set.

Characteristics		Control (n=210)	Intervention (n=202)	Total (N=412)
**Age (years)**				
	Median (range)	29.0 (16-59)	29.0 (17-58)	29.0 (16-59)
	IQR^a^	23.0-36.0	24.0-36.0	23.0-36.0
**Ethnic group^b^, n (%)**				
	European	149 (71.0)	148 (73.3)	297 (72.1)
	Māori (indigenous New Zealand)	57 (27.1)	56 (27.7)	113 (27.4)
	Asian	24 (11.4)	18 (8.9)	42 (10.2)
	Pasifika	21 (10.0)	21 (10.4)	42 (10.2)
	Other	3 (1.4)	4 (2.0)	7 (1.7)
	None	3 (1.4)	3 (1.5)	6 (1.5)
**Children^c^, n (%)**				
	One child or more	96 (45.7)	90 (44.6)	186 (45.1)
	No children	111 (52.9)	109 (54.0)	220 (53.4)
**Employment status, n (%)**				
	In paid employment	84 (40.0)	79 (39.1)	163 (39.6)
	Unemployed but looking for paid work	55 (26.2)	51 (25.2)	106 (25.7)
	Unemployed, not looking for paid work	10 (4.8)	8 (4.0)	18 (4.4)
	On a benefit	41 (19.5)	45 (22.3)	86 (20.9)
	Other	16 (7.6)	13 (6.4)	29 (7.0)
**Deprivation quintiles, n (%)**				
	1 (lowest deprivation)	21 (10.0)	21 (10.4)	42 (10.2)
	2	31 (14.8)	31 (15.3)	62 (15.0)
	3	36 (17.1)	45 (22.3)	81 (19.7)
	4	56 (26.7)	41 (20.3)	97 (23.5)
	5 (highest deprivation)	59 (28.1)	64 (31.7)	123 (29.9)
	Unknown	7 (3.0)	0 (0.0)	7 (1.7)
**Locality, n (%)**				
	Main urban area	174 (82.9)	169 (83.7)	343 (83.3)
	Other	29 (13.8)	33 (16.3)	62 (15.0)
**Partner relationship, n (%)**				
	Husband or wife	43 (20.5)	34 (16.8)	77 (18.7)
	Ex-husband or ex-wife	1 (0.5)	5 (2.5)	6 (1.5)
	Separated husband or wife	12 (5.7)	9 (4.5)	21 (5.1)
	Boyfriend or girlfriend	57 (27.1)	56 (27.7)	113 (27.4)
	Ex-boyfriend or ex-girlfriend	20 (9.5)	18 (8.9)	38 (9.2)
	De facto partner	60 (28.6)	69 (34.2)	129 (31.3)
	Ex-de facto partner	8 (3.8)	9 (4.5)	17 (4.1)
	Same sex partner	5 (2.4)	1 (0.5)	6 (1.5)
	Ex-same sex partner	4 (1.9)	0 (0.0)	4 (1.0)
**Length of partner relationship (years)**				
	Median (range)	4.0 (0.3-32.3)	4.0 (0.2-32.5)	4.0 (0.2-32.5)
	IQR	2.0-8.0	2.0-8.0	2.0-8.0
**Partner cohabiting, n (%)**				
	No	95 (45.2)	83 (41.1)	178 (43.2)
	Yes	111 (52.9)	116 (57.4)	227 (55.1)
**Plans for the relationship, n (%)**				
	End the relationship	57 (27.1)	43 (21.3)	100 (24.3)
	Remain in the relationship	57 (27.1)	45 (22.3)	102 (24.8)
	Unsure	96 (45.7)	114 (56.4)	210 (51.0)
**Where women accessed computer, n (%)**				
	My house	133 (63.3)	127 (62.9)	260 (63.1)
	My workplace	14 (6.7)	17 (8.4)	31 (7.5)
	A family or whanau member’s house	23 (11.0)	15 (7.4)	38 (9.2)
	A friend’s house	11 (5.2)	12 (5.9)	23 (5.6)
	Library	13 (6.2)	13 (6.4)	26 (6.3)
	Other	13 (6.2)	15 (7.4)	28 (6.8)
**Violence screening criteria (among four types of violence), n (%)**				
	1	58 (27.6)	52 (25.7)	110 (26.7)
	2-4	152 (72.4)	150 (74.3)	302 (73.3)
**Thoughts of self-harm^d^, n (%)**				
	No	200 (95.2)	188 (93.1)	388 (94.2)
	Yes	7 (3.3)	13 (6.4)	20 (4.9)
**Thoughts of suicide^d^, n (%)**				
	No	193 (91.9)	180 (89.1)	373 (90.5)
	Yes	13 (6.2)	21 (10.4)	34 (8.3)
**Depression (CESD-R^e,f^)**				
	Median (range)	31 (0-80)	34 (0-77)	32 (0-80)
	IQR	18-49	17·5-51.5	18-50
**Severity of violence (SVAWS^g,h^)**				
	Median (range)	85 (47-165)	84 (46-182)	84 (46-182)
	IQR	68-103	67-103.5	67.8-103.0

^a^IQR: interquartile range.

^b^Women could select one or more ethnicities.

^c^Children living in the household <18 years that woman cares for.

^d^CES-D items “wanted to hurt myself” or “wish I were dead” response “nearly every day for 2 weeks.”

^e^CESD-R: Center for Epidemiologic Studies Depression Scale, Revised.

^f^Raw scale scores without imputation. Higher scores indicate more depression symptoms; possible range 0-80).

^g^SVAWS: Severity of Violence Against Women Scale.

^h^Lower scores indicate lower exposure to violence; possible range: 46-196.

### Outcomes

Intervention estimates for primary outcomes across all time periods favored the intervention (Table 2) but were not statistically or clinically significant. The SVAWS 12-month adjusted intervention estimate was −2.47 (95% CI −7.95 to 3.02) and the CESD-R 1-month adjusted intervention estimate was −0.98 (95% CI −4.89 to 2.94). No study-related adverse events were reported. Intervention estimates for secondary outcomes were also not significant (see Multimedia Appendix 2).

The statistical analysis plan included subgroup analysis of primary outcomes by ethnicity (Māori vs non-Māori) and children (responsible for one or more child vs none). There was no differential intervention effect for violence or depression symptoms based on whether women were caring for children or not.

**Figure 3:**
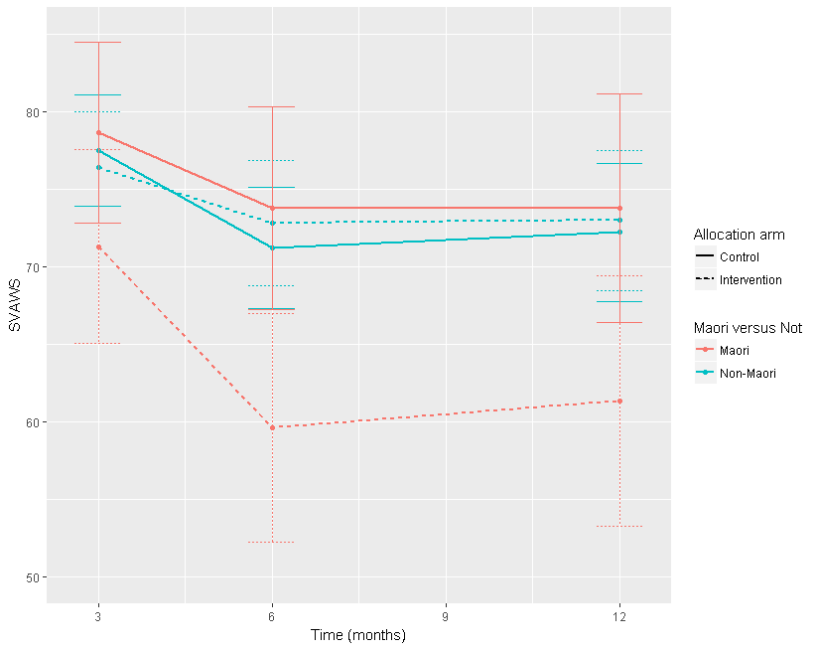
Severity of violence analysis by ethnicity and group. The Severity of Violence Against Women Scale (SVAWS) axis includes the postrandomization interquartile range values. The plotted data represent the estimated means of each group given a common starting baseline value, obtained from the fitted model.

There was a significant intervention effect for reducing violence for Māori women at 6 months (adjusted intervention estimate −14.19; 95% CI −24 to −4.37) and at 12 months (adjusted intervention estimate −12.44; 95% CI −23.35 to −1.54; Table 3 and Figure 3). There was also a significant intervention effect for reducing depression symptoms for Māori women at 3 months (adjusted intervention effect −8.7; 95% CI −15.9 to −1.6) but not at 6 or 12 months. Both violence (SVAWS) and depression (CESD-R) statistically significant changes exceeded standard error of measurement-based minimal clinically important differences [36] of 8 and 5, respectively.

**Table 2:** Primary outcomes.

Outcomes			Study group				Unadjusted		Adjusted^a^	
			Intervention		Control		Estimated intervention effect (95% CI)	*P* value	Estimated intervention effect (95% CI)	*P* value
			N	Mean (standard error)	N	Mean (standard error)				
**Severity of violence (SVAWS^b,c^)**										
	**3 months**									
		Total score	163	74.92 (2.10)	168	76.60 (2.07)	−1.67 (−7.48 to 4.13)	.47	−2.69 (−7.05 to 1.66)	.23
		Threat subscore	164	36.14 (1.05)	169	36.60 (1.00)	−0.46 (−3.32 to 2.39)	.64	−0.91 (−3.09 to 1.27)	.41
		Acts subscore	164	29.97 (0.93)	168	31.29 (0.97)	−1.32 (−3.96 to 1.33)	.14	−1.75 (−3.77 to 0.28)	.09
		Sexual aggression subscore^d^	163	8.93 (0.34)	169	8.70 (0.32)	0.23 (−0.69 to 1.14)	.53	0.95 (0.44-2.05)	.90
	**6 months**									
		Total score	162	69.36 (2.22)	180	70.88 (1.84)	−1.51 (−7.19 to 4.16)	.10	−2.16 (−7.06 to 2.73)	.39
		Threat subscore	162	32.38 (1.06)	181	33.51 (0.90)	−1.13 (−3.87 to 1.61)	.15	−1.45 (−3.90 to 1.00)	.25
		Acts subscore	162	28.49 (0.99)	180	29.27 (0.87)	−0.78 (−3.37 to 1.8)	.08	−0.96 (−3.18 to 1.26)	.40
		Sexual aggression subscore^d^	162	8.50 (0.35)	181	8.10 (0.26)	0.40 (−0.46 to 1.25)	.95	0.69 (0.32-1.48)	.34
	**12 months**									
		Total score	173	70.00 (2.16)	183	72.43 (2.12)	−2.43 (−8.39 to 3.53)	.26	−2.47 (−7.95 to 3.02)	.38
		Threat subscore	173	32.69 (1.08)	184	34.01 (1.03)	−1.32 (−4.25 to 1.62)	.22	−1.49 (−4.27 to 1.29)	.25
		Acts subscore	173	28.77 (0.93)	183	30.50 (1.01)	−1.73 (−4.43 to 0.97)	.10	−1.57 (−4.01 to 0.87)	.21
		Sexual aggression subscore^d^	173	8.54 (0.33)	184	8.01 (0.28)	0.53 (−0.32 to 1.38)	.44	1.09 (0.51-2.35)	.83
**Depression (CESD-R^e,f^)**										
	3 months		165	25.69 (1.52)	167	27.04 (1.56)	−1.35 (−5.64 to 2.93)	.57	−1.85 (−5.49 to 1.8)	.32
	6 months		162	23.68 (1.65)	181	24.27 (1.45)	−0.59 (−4.9 to 3.73)	.53	−1.56 (−5.24 to 2.11)	.40
	12 months		172	22.59 (1.63)	184	23.30 (1.51)	−0.71 (−5.08 to 3.66)	.51	−0.98 (−4.89 to 2.94)	.63

^a^All adjusted results are adjusted for baseline value of the respective score; SVAWS threat subscore was also adjusted for age group. SVAWS sexual aggression subscore and CESD-R were also adjusted for children.

^b^SVAWS: Severity of Violence Against Women Scale.

^c^Lower scores indicate lower exposure to violence.

^d^dSVAWS sexual aggression subscore was dichotomized for the adjusted analyses (low=0; high>0), and the adjusted treatment effects for this outcome are odds ratios. The full sexual aggression subscores were retained for the unadjusted analyses. CIs are based on a t-distribution using Welch approximate degrees of freedom (allowing for different variances between groups). All analyses have been carried out in the intention-to-treat analysis set. *P* values are based on Mann-Whitney test.

^e^CESD-R: Center for Epidemiologic Studies Depression Scale, Revised.

^f^Raw scale scores without imputation. Higher scores indicate more depression symptoms; possible range 0-80).

^g^SVAWS: Severity of Violence Against Women Scale.

^h^Higher scores indicate more depression symptoms.

**Table 3:** Primary outcomes subgroup analysis.

Outcomes per subgroup				Estimated intervention effect (95% CI)	*P* value
**Severity of violence (SVAWS**^a^**)**					
	**Ethnicity**				
		**3 Months**			
			Non-Maori	−1.09 (−6.16 to 3.98)	.67
			Maori	−7.35 (−15.84 to 1.15)	.09
		**6 Months**			
			Non-Maori	1.59 (−4.02 to 7.19)	.58
			Maori	−14.19 (−24 to −4.37)	.005
		**12 Months**			
			Non-Maori	0.76 (−5.57 to 7.09)	.81
			Maori	−12.44 (−23.35 to −1.54)	.03
	**Children**				
		**3 Months**			
			No Children	−2.91 (−8.8 to 2.98)	.33
			Children	−2.70 (−9.24 to 3.85)	.42
		**6 Months**			
			No Children	−3.03 (−9.57 to 3.51)	.36
			Children	−1.31 (−8.76 to 6.14)	.73
		**12 Months**			
			No Children	−3.1 (−10.62 to 4.4)	.42
			Children	−1.65 (−9.85 to 6.55)	.69
**Depression (CESD-R**^b,c^**)**					
	**Ethnicity**				
		**3 Months**			
			Non-Maori	0.56 (−3.67 to 4.79)	.80
			Maori	−8.73 (−15.88 to −1.58)	.02
		**6 Months**			
			Non-Maori	−0.51 (−4.74 to 3.72)	.81
			Maori	−4.55 (−11.97 to 2.87)	.23
		**12 Months**			
			Non-Maori	1.36 (−3.16 to 5.88)	.56
			Maori	−7.75 (−15.57 to 0.07)	.05
	**Children**				
		**3 Months**			
			No Children	−0·65 (−5.55 to 4.24)	.79
			Children	−1.79 (−6.7 to 3.13)	.48
		**6 Months**			
			No Children	−3.00 (−8.32 to 2.32)	.27
			Children	−3.31 (−8.78 to 2.16)	.24
		**12 Months**			
			No Children	−1.25 (−6.84 to 4.33)	.66
			Children	1.24 (−4.55 to 7.04)	.67

^a^SVAWS: Severity of Violence Against Women Scales.

^b^CESD-R: Center for Epidemiologic Studies Depression Scale, Revised.

^c^Subgroup estimates were obtained through fitting a model including an interaction between allocation arm and Māori or children. Intervention effects are adjusted for baseline values. All analyses have been carried out in the intention-to-treat analysis set.

## Discussion

We tested a confidential Web-based safety decision aid (*isafe*) for women experiencing IPV, a complex health and social problem. Informed by an empowerment model [9] and decision-aid science [10], the complex intervention included three interactive components: (1) priority setting for decision making, (2) risk assessment, and (3) creation of a personalized safety action plan. In our RCT in a general New Zealand population of women 16 years of age and older, we discovered access to the regionalized *isafe* intervention was effective in reducing violence at 6 and 12 months and in reducing depression symptoms at 3 months—limited to indigenous Māori women. This is an important finding given the overrepresentation of Māori in New Zealand family violence statistics. Māori women’s rate of physical and or sexual IPV in the past 12 months (14.1%) is more than three times higher than for New Zealand European women (3.9%) [37]. In addition, as in other colonized nations [38], indigenous Māori experience significant health inequities, including higher levels of unmet needs [39]. A treatment effect in a population group that experiences significant health disparities is a welcome, important finding in the struggle and moral obligation to reduce health inequities.

We offer two possible explanations for the mechanism of action for the interaction between ethnicity and outcomes. Colonization and assimilation significantly disrupted traditional Māori whānau structures and the complementary roles of men and women [40,41]. The resultant disablement of cultural practices that kept women and children safe means for many, the transmission of violence across generations has become normalized. One explanation for our findings, therefore, is that the normalization of violence in Māori whānau (families) blinds women to their risk. Completion of the *isafe* intervention brings to women’s attention the level of danger they and their children are living with and empowers them to consider their priorities for safety, making them amenable to the intervention. Another explanation is that Māori women experience racism and discrimination when accessing social, health, and justice services [42]; often avoid accessing services because of the associated discrimination and other socioeconomic determinants such as cost; and are more likely, compared with non-Māori, to report unmet needs for health care [43]. As a culturally inclusive, fully automated Web-based intervention study [12-14], *isafe* avoids (but not eliminates) the potential for racism or discrimination, enabling women to access help without fear of judgment or having their children removed. Māori women may have appreciated that *isafe* was culturally inclusive rather than perpetuating the myth that IPV is only a Māori problem. This is consistent with advice from aboriginal women interviewed about addressing the problem of fetal alcohol spectrum disorders: mainstream educational strategies should be inclusive of the aboriginal community but not target them [44]. This needs to be balanced with support and advocacy for community-led Kaupapa Māori interventions by Māori for Māori [45]. This has important implications for future implementation of *isafe*.

Secondary analyses of Māori subgroup data to confirm the profile for Māori women using *isafe* (ie, does the study include Māori women at highest risk) and exploring multiple ethnicities and Māori ancestry is needed. For example, in the 2013 New Zealand census, among those who identified as Māori, half (54%) reported additional ethnicities (higher proportion for younger persons) [46]. We acknowledge that while our estimates of *isafe* impact for Māori women are significant, they are imprecise, with wide confidence intervals. The limited number of Māori participants (n=113) and essentialism in dichotomizing ethnicity in this RCT are two design constraints limiting our knowing when, for whom, and in what ways *isafe* might be beneficial to women. Alongside RCTs, research approaches such as critical realism are needed to better understand and test complex interventions for complex health and social problems such as IPV [44]. Family violence often cooccurs with a range of social and health challenges. Future work might extend the safety decision aid to address violence-related health concerns, or “join up” with other health, social, and family violence prevention initiatives.

In considering whether *isafe* is a promising intervention for a general population of women, several study limitations are important to consider. By design, all women completed baseline violence and mental health measurements (eg, SVAWS and WEB). Engaging with these measurement items may have raised women’s awareness of the violence in their relationships and muted an intervention effect. In addition, outcomes were self-reported, introducing common method bias. There also may be important intermediate outcomes that we did not measure. For example, the Australian iDecide [47] and Canadian iCan [48] safety decision aid trials include general self-efficacy and self-efficacy for safety planning, respectively, in their suite of outcome measures. These two trials have not yet reported their outcomes. Finally, given the importance of considering patient-centered trial acceptability [49], women in both the intervention and control groups reported that *isafe* was useful. We had added five participant-centered acceptability questions at the 12-month follow-up midway through our trial. Among the subset of 215 women who completed their 12-month follow-up assessment after October 14, 2014, the majority agreed (true or somewhat true) the *isafe* study “provided me with new skills” (78%), “provided me with useful information” (91%), “I would suggest the site to others” (90%), “I enjoyed visiting *isafe* ” (87%), and an open-ended question, “Overall, how did you find participating in the *isafe* study?” As one woman in the control group shared, *I felt protected & secure with opening up about my situation. Not judged, just helped.* Importantly, there were no differences in *isafe* acceptability by ethnicity (Māori or non-Māori). Our usefulness data suggests that although non-Māori women in the study failed to experience reduced violence or improved mental health, they did find it useful. This signals that further work is needed in understanding women’s pathways to health and safety, as well as developing woman-centered measures of potential eHealth safety decision aid benefits.

This trial is one of several studies testing a Web-based safety decision aid for women experiencing IPV. Only the first trial, conducted in four US states [50], has published their findings. Among 725 consenting participants enrolled through a telephone conversation with a trained research assistant, 672 (93%) completed the 12-month assessment. Our 12-month retention rate of 87% is encouraging for future studies preferring fully automated Web-based registration. We focused our limited personal retention contacts on the 12-month assessment, particularly for 35 women who had not been automatically contacted for 3- and 6-month assessments because of a technology error. Among the three primary outcomes in the US trial, decisional conflict reduced for women in the intervention group to a greater degree than women in the control groups between the baseline pre- and baseline posttest; there were no significant differences at 6 or 12 months. The proportion of safety behaviors perceived as helpful increased 12% in the intervention group (baseline to 12 months) compared with 9% in the control group (*P*=.037). There were no differences between groups in the reduction of violence (or depression symptoms) over time in the US trial. Our *isafe* exploratory (not in our a priori statistical analysis plan) analysis of secondary outcomes by ethnicity suggests a larger reduction in decisional conflict among Māori women compared with non-Māori women at 3 months, but this was not statistically significant. The results are mixed in regard to safety behaviors. In particular, the Safety Behavior Checklist Helpfulness score, which resembles the measure on which a positive result was found in the US trial, shows a moderate signal favoring the control arm (on the order of 10% for Māori and 3% for non-Māori). The safety behavior activities and “what works” for women in New Zealand, particularly for Māori women, may be different compared with the United States. Further research is needed to better understand women’s safety behaviors and potentially improved measurement instruments [51].

No adverse events were recorded in either the *isafe* or US trial. The *isafe* trial supports a differential effect of the Web-based safety decision aid for indigenous Māori women. As the evidence accumulates across the four current (and possibly future) international Web-based safety decision aid trials [47,48,50,52], ethical [53,54], methodological [54,55], theoretical [12,52], and practice knowledge will be gained. Importantly, these 4 studies are all examining the same intervention (with regionalization), measuring common outcomes, with follow-up of at least 12 months. This evidence will allow us to identify the magnitude of benefit and the specific populations amenable to interventions [6]. In addition to indigenous women, the safety decision aid may benefit other populations of at risk women.

*Isafe* was found to be a valuable early intervention resource for Māori women experiencing IPV. Dissemination of *isafe* nationwide has the potential to contribute to reducing IPV recurrence, improving mental health, and reducing injuries and perhaps femicide amongst Māori women exposed to IPV. In addition, *isafe* is likely to be a cost effective, economically sustainable resource. In 2014, family violence was conservatively estimated to cost New Zealand NZ $4.1 billion per annum, with NZ $2,198 in costs avoided for every New Zealander whose experience of family violence is prevented [56]. Cost analyses are needed to calculate the cost per woman using the decision aid against the yearly cost of maintaining advocacy and counseling services. In developing a sustainable open access online platform for *isafe*, ongoing modification to keep pace with technology advancements, people’s use of technology, and future evidence will be needed. *isafe* may also be a useful resource to include in formal health [4], police, and justice IPV responses to women experiencing violence, alongside the necessary services working with abusive partners [57]. Dissemination studies using the Reach, Effectiveness, Adoption, Implementation, and Maintenance framework [58] for example, are needed to guide evidence-based practice across these intervention locations.

In conclusion, the interactive Web-based *isafe* decision aid includes risk assessment, priority setting for decision making, and creation of a personalized safety action plan. Our findings provide impetus for further dissemination and testing of interactive, individualized Web-based interventions to reduce IPV and associated health harms in at risk populations.

## Appendix

Multimedia Appendix 1 Outcome assessment schedule for the *isafe* trial.

Multimedia Appendix 2 Secondary outcomes.

Multimedia Appendix 3 CONSORT‐EHEALTH checklist (V 1.6.1).

## Abbreviations

CESD-R: Center for Epidemiologic Studies Depression Scale-Revised
DA: Danger Assessment
DA-R: Danger Assessment-Revised
DMC: data monitoring committee
eHealth: electronic health
IPV: intimate partner violence
ITT: intention-to-treat
IQR: interquartile range
RCT: randomized controlled trial
SVAWS: Severity of Violence Against Women Scale
WEB: Women’s Experience with Battering

## References

[1] Campbell JC (2002). Health consequences of intimate partner violence. Lancet.

[2] Garcia-Moreno C, DeVries K, Stockl H, Pallitto C, Watts C, Abrahams N (2013). Global and regional estimates of violence against women: Prevalence and health effects of intimate partner violence and non-partner sexual violence.

[3] Lim SS, Vos T, Flaxman AD, Danaei G, Shibuya K, Adair-Rohani H, Amann M, Anderson HR, Andrews KG, Aryee M, Atkinson C, Bacchus LJ, Bahalim AN, Balakrishnan K, Balmes J, Barker-Collo S, Baxter A, Bell ML, Blore JD, Blyth F, Bonner C, Borges G, Bourne R, Boussinesq M, Brauer M, Brooks P, Bruce NG, Brunekreef B, Bryan-Hancock C, Bucello C, Buchbinder R, Bull F, Burnett RT, Byers TE, Calabria B, Carapetis J, Carnahan E, Chafe Z, Charlson F, Chen H, Chen JS, Cheng AT, Child JC, Cohen A, Colson KE, Cowie BC, Darby S, Darling S, Davis A, Degenhardt L, Dentener F, Des Jarlais DC, Devries K, Dherani M, Ding EL, Dorsey ER, Driscoll T, Edmond K, Ali SE, Engell RE, Erwin PJ, Fahimi S, Falder G, Farzadfar F, Ferrari A, Finucane MM, Flaxman S, Fowkes FG, Freedman G, Freeman MK, Gakidou E, Ghosh S, Giovannucci E, Gmel G, Graham K, Grainger R, Grant B, Gunnell D, Gutierrez HR, Hall W, Hoek HW, Hogan A, Hosgood HD, Hoy D, Hu H, Hubbell BJ, Hutchings SJ, Ibeanusi SE, Jacklyn GL, Jasrasaria R, Jonas JB, Kan H, Kanis JA, Kassebaum N, Kawakami N, Khang Y, Khatibzadeh S, Khoo J, Kok C, Laden F, Lalloo R, Lan Q, Lathlean T, Leasher JL, Leigh J, Li Y, Lin JK, Lipshultz SE, London S, Lozano R, Lu Y, Mak J, Malekzadeh R, Mallinger L, Marcenes W, March L, Marks R, Martin R, McGale P, McGrath J, Mehta S, Mensah GA, Merriman TR, Micha R, Michaud C, Mishra V, Mohd HK, Mokdad AA, Morawska L, Mozaffarian D, Murphy T, Naghavi M, Neal B, Nelson PK, Nolla JM, Norman R, Olives C, Omer SB, Orchard J, Osborne R, Ostro B, Page A, Pandey KD, Parry CD, Passmore E, Patra J, Pearce N, Pelizzari PM, Petzold M, Phillips MR, Pope D, Pope CA, Powles J, Rao M, Razavi H, Rehfuess EA, Rehm JT, Ritz B, Rivara FP, Roberts T, Robinson C, Rodriguez-Portales JA, Romieu I, Room R, Rosenfeld LC, Roy A, Rushton L, Salomon JA, Sampson U, Sanchez-Riera L, Sanman E, Sapkota A, Seedat S, Shi P, Shield K, Shivakoti R, Singh GM, Sleet DA, Smith E, Smith KR, Stapelberg NJC, Steenland K, Stöckl H, Stovner LJ, Straif K, Straney L, Thurston GD, Tran JH, Van Dingenen R, van Donkelaar A, Veerman JL, Vijayakumar L, Weintraub R, Weissman MM, White RA, Whiteford H, Wiersma ST, Wilkinson JD, Williams HC, Williams W, Wilson N, Woolf AD, Yip P, Zielinski JM, Lopez AD, Murray CJ, Ezzati M, AlMazroa MA, Memish ZA (2012). A comparative risk assessment of burden of disease and injury attributable to 67 risk factors and risk factor clusters in 21 regions, 1990-2010: a systematic analysis for the Global Burden of Disease Study 2010. Lancet.

[4] García-Moreno C, Hegarty K, d'Oliveira AF, Koziol-McLain J, Colombini M, Feder G (2015). The health-systems response to violence against women. The Lancet.

[5] World Health Organization (2016). Global plan of action to strengthen the role of the health system within a national multisectoral response to address interpersonal violence, in particular against women and girls, and against children.

[6] Rivas C, Ramsay J, Sadowski L, Davidson LL, Dunne D, Eldridge S, Hegarty K, Taft A, Feder G (2015). Advocacy interventions to reduce or eliminate violence and promote the physical and psychosocial well-being of women who experience intimate partner abuse. Cochrane Database Syst Rev.

[7] Fanslow JL, Robinson EM (2010). Help-seeking behaviors and reasons for help seeking reported by a representative sample of women victims of intimate partner violence in New Zealand. J Interpers Violence.

[8] Glass N, Eden KB, Bloom T, Perrin N (2009). Computerized aid improves safety decision process for survivors of intimate partner violence. J Interpers Violence.

[9] Dutton MA (1992). Empowering and healing the battered woman: a model for assessment and intervention.

[10] Stacey D, Légaré F, Col NF, Bennett CL, Barry MJ, Eden KB, Holmes-Rovner M, Llewellyn-Thomas H, Lyddiatt A, Thomson R, Trevena L, Wu JH (2014). Decision aids for people facing health treatment or screening decisions. Cochrane Database Syst Rev.

[11] Eden KB, Perrin NA, Hanson GC, Messing JT, Bloom TL, Campbell JC, Gielen AC, Clough AS, Barnes-Hoyt JS, Glass NE (2015). Use of online safety decision aid by abused women. Am J Prev Med.

[12] Young-Hauser AM, Eden KB, Wilson D, Koziol-Mclain J (2014). Intimate partner violence: modifying an internet-based safety decision aid to a New Zealand context. J Technol Hum Serv.

[13] Koziol-McLain J, Vandal AC, Nada-Raja S, Wilson D, Glass NE, Eden KB, McLean C, Dobbs T, Case J (2015). A web-based intervention for abused women: the New Zealand
*isafe* randomised controlled trial protocol. BMC Public Health.

[14] Koziol-McLain J, McLean C, Rohan M, Sisk R, Dobbs T, Nada-Raja S, Wilson D, Vandal AC (2016). Participant recruitment and engagement in automated eHealth trial registration: challenges and opportunities for recruiting women who experience violence. J Med Internet Res.

[15] Saaty T (2008). Decision making with the analytic hierarchy process. IJSSCI.

[16] Campbell JC, Webster DW, Glass N (2009). The danger assessment: validation of a lethality risk assessment instrument for intimate partner femicide. J Interpers Violence.

[17] Glass N, Perrin N, Hanson G, Bloom T, Gardner E, Campbell JC (2008). Risk for reassault in abusive female same-sex relationships. Am J Public Health.

[18] Eaton WW, Smith C, Ybarra M, Muntaner C, Tien A, Maruish ME (2004). Center for Epidemiologic Studies Depression Scale: review and revision (CESD and CESD-R). The Use Of Psychological Testing For Treatment Planning And Outcomes third Edition, Volume 3.

[19] Radloff LS (2016). The CES-D Scale: a self-report depression scale for research in the general population. Appl Psychol Meas.

[20] Van Dam NT, Earleywine M (2011). Validation of the Center for Epidemiologic Studies Depression Scale--Revised (CESD-R): pragmatic depression assessment in the general population. Psychiatry Res.

[21] Marshall L (1992). Development of the severity of violence against women scales. J Fam Viol.

[22] Smith PH, Earp JA, DeVellis R (1995). Measuring battering: development of the Women's Experience with Battering (WEB) Scale. Womens Health.

[23] Blanchard EB, Jones-Alexander J, Buckley TC, Forneris CA (1996). Psychometric properties of the PTSD Checklist (PCL). Behav Res Ther.

[24] Norris FH, Hamblen JL, Wilson JP, Keane TM (2004). Standardized self-report measures of civilian trauma and PTSD. Assessing Psychological Trauma and PTSD, 2nd ed.

[25] Reinert DF, Allen JP (2007). The alcohol use disorders identification test: an update of research findings. Alcohol Clin Exp Res.

[26] Saunders JB, Aasland OG, Babor TF, de la Fuente JR, Grant M (1993). Development of the Alcohol Use Disorders Identification Test (AUDIT): WHO collaborative project on early detection of persons with harmful alcohol consumption--II. Addiction.

[27] Smith PC, Schmidt SM, Allensworth-Davies D, Saitz R (2010). A single-question screening test for drug use in primary care. Arch Intern Med.

[28] Yudko E, Lozhkina O, Fouts A (2007). A comprehensive review of the psychometric properties of the Drug Abuse Screening Test. J Subst Abuse Treat.

[29] O'Connor AM (1995). Validation of a decisional conflict scale. Med Decis Making.

[30] Sullivan CM, Bybee DI (1999). Reducing violence using community-based advocacy for women with abusive partners. J Consult Clin Psychol.

[31] Parker B, McFarlane J, Soeken K, Silva C, Reel S (1999). Testing an intervention to prevent further abuse to pregnant women. Res Nurs Health.

[32] White IR, Horton NJ, Carpenter J, Pocock SJ (2011). Strategy for intention to treat analysis in randomised trials with missing outcome data. Br Med J.

[33] White IR, Thompson SG (2005). Adjusting for partially missing baseline measurements in randomized trials. Stat Med.

[34] Carpenter J, Kenward M (2007). Missing data in randomised controlled trials - a practical guide.

[35] Bates D, Mächler M, Bolker B, Walker S (2015). Fitting Linear Mixed-Effects Models Using lme4. J Stat Soft.

[36] Wyrwich KW, Nienaber NA, Tierney WM, Wolinsky FD (1999). Linking clinical relevance and statistical significance in evaluating intra-individual changes in health-related quality of life. Med Care.

[37] Fanslow J, Robinson E, Crengle S, Perese L (2010). Juxtaposing beliefs and reality: prevalence rates of intimate partner violence and attitudes to violence and gender roles reported by New Zealand women. Violence Against Women.

[38] Kirmayer LJ, Brass G (2016). Addressing global health disparities among Indigenous peoples. Lancet.

[39] Ministry of Health (2013). The health of Māori adults and children.

[40] Kruger T, Pitman M, Grennell D, McDonald T, Mariu D, Pomare A (2004). Transforming Whanau Violence: A Conceptual Framework (2nd ed).

[41] Dobbs T, Eruera M (2014). Kaupapa Māori wellbeing framework: the basis for whānau violence prevention and intervention.

[42] Harris R, Tobias M, Jeffreys M, Waldegrave K, Karlsen S, Nazroo J (2006). Effects of self-reported racial discrimination and deprivation on Māori health and inequalities in New Zealand: cross-sectional study. Lancet.

[43] Ministry of Health (2016). Annual update of key results 2015/2016: New Zealand Health Survey.

[44] Blagg H, Bluett-Boyd N, Williams E (2015). Innovative models in addressing violence against Indigenous women: state of knowledge paper.

[45] Smith L (1999). Decolonizing methodologies: research and indigenous peoples.

[46] Statistics New Zealand (2013). Archive.stats.

[47] Hegarty K, Tarzia L, Murray E, Valpied J, Humphreys C, Taft A, Gold L, Glass N (2015). Protocol for a randomised controlled trial of a web-based healthy relationship tool and safety decision aid for women experiencing domestic violence (I-DECIDE). BMC Public Health.

[48] Ford-Gilboe M, Varcoe C, Scott-Storey K, Wuest J, Case J, Currie LM, Glass N, Hodgins M, MacMillan H, Perrin N, Wathen CN (2017). A tailored online safety and health intervention for women experiencing intimate partner violence: the iCAN Plan 4 Safety randomized controlled trial protocol. BMC Public Health.

[49] Kost RG, Lee LM, Yessis J, Wesley RA, Henderson DK, Coller BS (2013). Assessing participant-centered outcomes to improve clinical research. N Engl J Med.

[50] Glass NE, Perrin NA, Hanson GC, Bloom TL, Messing JT, Clough AS, Campbell JC, Gielen AC, Case J, Eden KB (2017). The longitudinal impact of an internet safety decision aid for abused women. Am J Prev Med.

[51] Wilson D, Jackson D, Herd R (2016). Confidence and connectedness: Indigenous Māori women's views on personal safety in the context of intimate partner violence. Health Care Women Int.

[52] Tarzia L, Murray E, Humphreys C, Glass N, Taft A, Valpied J, Hegarty K (2016). I-DECIDE: an online intervention drawing on the Psychosocial Readiness Model for women experiencing domestic violence. Womens Health Issues.

[53] Allenby R, Dobbs T, Diesfeld K, Nada Raja S, Wilson D, Koziol-McLain J (2016). Safety in online research with women experiencing intimate partner violence: what about the children?. Ethics Behav.

[54] Tarzia L, Valpied J, Koziol-McLain J, Glass N, Hegarty K (2017). Methodological and ethical challenges in a web-based randomized controlled trial of a domestic violence intervention. J Med Internet Res.

[55] Ford-Gilboe M, Wathen CN, Varcoe C, MacMillan HL, Scott-Storey K, Mantler T, Hegarty K, Perrin N (2016). Development of a brief measure of intimate partner violence experiences: the Composite Abuse Scale (Revised)-Short Form (CASR-SF). BMJ Open.

[56] Kahui S, Snively S (2014). Measuring the Economic Costs of Child Abuse and Intimate Partner Violence to New Zealand.

[57] Family Violence Death Review Committee (2017). Fifth Report Data: January 2009 to December 2015.

[58] Gaglio B, Shoup JA, Glasgow RE (2013). The RE-AIM framework: a systematic review of use over time. Am J Public Health.

