# Supplementary material for: Efficacy of a Web-Based Safety Decision Aid for Women Experiencing Intimate Partner Violence: Randomized Controlled Trial
Source: J Med Internet Res. 2018 Jan 10;19(12):e426. doi: 10.2196/jmir.8617 (PMC6858022; doi:10.2196/jmir.8617)
Supplement: Multimedia Appendix 1 [file jmir_v19i12e426_app1.pdf]

Supplementary Table: Outcome assessment schedule for the isafe trial

Outcome	Higher/lower scores beneficial?	Time point			
		BL	3m	6m	12m
Exposure to violence domain					
Severity of Violence Against Women Scale (SVAWS)	Lower	X	X	X	X*
SVAWS Threats of violence subscore	Lower	X	X	X	X
SVAWS Acts of violence subscore	Lower	X	X	X	X
SVAWS Sexual violence subscore	Lower	X	X	X	X
Women's Experience with Battering (WEB)	Lower	X		X	X
Mental health domain					
Center for Epidemiologic Studies Depression scale, Revised	Lower	X	X	X	X*
Post-Traumatic Stress Disorder Checklist, Civilian Version	Lower	X**	X**	X**	X
Alcohol Use Disorder Identification Test (AUDIT) dichotomised	Lower	X		X	X
Drug Abuse Screening Tool (DAST-10)	Lower	X		X	X
Decisional process domain					
Decisional Conflict Scale (DCS)	Lower	X	X	X	X
Safety-seeking behaviour domain					
Safety Checklist	Higher	X	X	X	X
Safety Checklist Helpfulness	Higher	X	X	X	X

Notes: BL: Baseline; *: Primary endpoint; **: One item missing in a majority of cases
