# Supplementary material for: Efficacy of a Web-Based Safety Decision Aid for Women Experiencing Intimate Partner Violence: Randomized Controlled Trial
Source: J Med Internet Res. 2018 Jan 10;19(12):e426. doi: 10.2196/jmir.8617 (PMC6858022; doi:10.2196/jmir.8617)
Supplement: Multimedia Appendix 2 [file jmir_v19i12e426_app2.pdf]

Supplementary Table: Secondary outcomes	Study Group						Unadjusted			Adjusted[*]		
	Intervention			Control			Estimated intervention effect (95% CI)		*P* value	Estimated intervention effect (95% CI)		*P* value
	N	Mean (SE)		N	Mean (SE)							
Womens' Experience with Battering (WEB)												
6 Months	162	36.88	(1.28)	181	38.13	(1.15)	-1.24	(-4.62,2.14)	0.51	0.45	(0.1,1.84)	0.24
12 Months	173	36.04	(1.29)	184	36.64	(1.21)	-0.60	(-4.09,2.89)	0.77	0.63	(0.19,2.19)	0.43
Post-Traumatic Stress Disorder Checklist, Civilian Version (PCL-C)												
6 Months	108	38.83	(1.61)	123	40.86	(1.58)	-2.03	(-6.48,2.42)	0.40	-1.61	(-5.29,2.07)	0.39
12 Months	167	38.35	(1.26)	175	39.48	(1.29)	-1.13	(-4.68,2.43)	0.62	-1.72	(-4.71,1.26)	0.26
Alcohol Use Disorder Identification Test (AUDIT) dichotomized[a]												
6 Months	135	7.23	(0.68)	149	7.31	(0.59)	-0.08	(-1.86,1.7)	0.59	0.39	(0.12,1.09)	0.08
12 Months	148	6.70	(0.56)	163	6.85	(0.59)	-0.14	(-1.75,1.46)	0.74	1.25	(0.44,3.52)	0.66
Drug Abuse Screening Tool (DAST-10)[b]												
6 Months	63	3.87	(0.28)	76	3.82	(0.26)	0.06	(-0.7,0.81)	0.72	0.81	(0.2,3.25)	0.75
12 Months	70	3.34	(0.26)	78	3.27	(0.26)	0.07	(-0.65,0.8)	0.54	3.66	(0.85,20.86)	0.1
Decisional Conflict Scale (DCS)												
Post-Baseline	196	47.18	(1.08)	203	45.40	(1.03)	1.77	(-1.17,4.72)	0.16	0.05	(-2.73,2.83)	0.97
3 Months	164	14.23	(1.34)	169	14.98	(1.32)	-0.75	(-4.44,2.94)	0.71	-1.09	(-3.96,1.77)	0.46
6 Months	161	12.22	(1.26)	181	12.14	(1.27)	0.07	(-3.44,3.58)	0.61	-0.76	(-3.61,2.09)	0.6
12 Months	172	9.56	(1.07)	183	9.30	(1.04)	0.26	(-2.69,3.2)	0.98	-0.43	(-3.23,2.37)	0.76
Safety Checklist												
3 Months	165	7.64	(0.34)	169	7.60	(0.34)	0.03	(-0.91,0.98)	0.89	-0.24	(-1.05,0.57)	0.56
6 Months	162	7.73	(0.36)	181	7.46	(0.34)	0.27	(-0.7,1.24)	0.53	0.09	(-0.77,0.96)	0.83
12 Months	165	7.64	(0.34)	169	7.60	(0.34)	0.03	(-0.91,0.98)	0.89	0.30	(-0.58,1.18)	0.5
Safety Checklist Helpfulness												
3 Months	161	13.91	(2.02)	165	13.64	(1.88)	-0.27	(-5.16,5.71)	0.97	0.91	(0.45,1.82)	0.78
6 Months	159	13.25	(1.89)	177	13.01	(1.74)	-0.24	(-4.83,5.3)	0.77	1.15	(0.58,2.3)	0.69
12 Months	169	14.81	(2.06)	178	13.61	(1.8)	-1.20	(-4.18,6.57)	0.94	0.87	(0.44,1.71)	0.69
